# Impact of Intraoperative Albumin Use During Lung Transplantation on Primary Graft Dysfunction

**DOI:** 10.3390/jcm14217843

**Published:** 2025-11-05

**Authors:** Yoshio Tatsuoka, Krzysztof J. Zembrzuski, Jake G. Natalini, Stephanie H. Chang, Jennie Y. Ngai

**Affiliations:** 1 Department of Anesthesiology, Perioperative Care, and Pain Medicine, New York University Langone Health, New York, NY 10016, USA; 2 Division of Pulmonary, Critical Care, and Sleep Medicine, Department of Medicine, New York University Langone Health, New York, NY 10016, USA; 3 Department of Cardiothoracic Surgery, New York University Langone Health, New York, NY 10016, USA

**Keywords:** lung transplantation, primary graft dysfunction, albumin

## Abstract

**Background:** Primary graft dysfunction (PGD) is the leading cause of early mortality after lung transplantation. Albumin is commonly used during lung transplantation to maintain intravascular volume while minimizing total intravenous fluid administration, given the established association between larger intravenous fluid and PGD. However, the direct impact of albumin on PGD remains unclear. **Methods:** We conducted a single-center retrospective cohort study of lung transplant recipients between 2018 and 2023. We calculated the corrected albumin proportion (cAP), representing the ratio of albumin to total intravenous fluid administered. We analyzed associations between cAP and PGD at 24, 48, and 72 h, as well as secondary outcomes including total fluid administration, 30-day acute kidney injury, mortality, and ICU length of stay. **Results:** A total of 190 patients were included in this study. A higher cAP was associated with lower total intravenous fluid administration (*r* = −0.15, *p* = 0.03), whereas a higher total intravenous fluid administration was associated with higher PGD at 72 h (OR 1.02, 95% CI 1.00–1.03, *p* = 0.04). However, cAP was not independently associated with PGD or other short-term outcomes. **Conclusions:** Intraoperative albumin use modestly reduced total intravenous fluid administration but was not independently associated with significant reductions in PGD or improvements in other short-term outcomes.

## 1. Introduction

Lung transplantation remains the definitive treatment for patients with end-stage lung disease. Despite substantial improvements in perioperative management and immunosuppression, lung transplantation continues to have the highest mortality rate among solid organ transplants [1]. Primary graft dysfunction (PGD), a form of acute lung injury occurring within the first 72 h after transplantation, remains the leading cause of early morbidity and mortality [2]. It is defined and graded from 0 (no PGD) to 3 (the most severe PGD) based on the ratio of partial pressure of arterial oxygen to the fraction of inspired oxygen (P/F ratio) and chest X-ray findings, using standardized criteria from the International Society for Heart and Lung Transplantation [3]. PGD reflects ischemia–reperfusion injury with early innate immune response and increased pulmonary capillary permeability, leading to pulmonary edema and hypoxemia [4]. Accordingly, prevention of PGD remains a major focus of intraoperative and early postoperative management. One potential modifiable risk factor for PGD is perioperative intravenous fluid administration. Multiple observational studies have demonstrated the association between larger intraoperative intravenous fluid administration and increased PGD incidence [5,6,7]. Albumin, a colloid solution with prolonged intravascular half-life and potential antioxidative and anti-inflammatory effects, is frequently substituted for crystalloids during cardiothoracic surgery [8,9]. Theoretically, albumin could achieve hemodynamic stability while minimizing interstitial lung fluid accumulation. However, the clinical relevance of these theoretical effects remains controversial. Some studies have shown reduced fluid requirements with albumin administration, whereas others have reported no improvement in postoperative pulmonary or renal outcomes [9,10,11,12,13]. Importantly, the direct relationship between intraoperative albumin use during lung transplantation and PGD has not been systematically examined. This study aimed to evaluate the association between intraoperative albumin administration and the incidence of PGD. To quantify albumin exposure, we introduced a corrected albumin proportion (cAP) that accounts for differences in albumin concentration and total intravenous fluid volume. We hypothesized that a higher cAP would be associated with lower total fluid administration and reduced incidence of PGD.

## 2. Materials and Methods

Study Design: This retrospective, single-center cohort study was approved by the Institutional Review Board (IRB) of New York University Langone Hospital, New York, NY, USA, with informed consent waived (IRB #i24-01247). Adult patients (≥18 years) undergoing unilateral or bilateral lung transplantation from November 2018 to January 2023 were included. Exclusion criteria were retransplantation, dual-organ transplantation (e.g., heart-lung transplantation), simultaneous surgical procedures (e.g., cardiac valve replacement), and incomplete intraoperative data.

Data Collection and Variable Calculation: We identified patients who met the study criteria and manually reviewed individual charts to collect perioperative data. Given that the total volume of albumin and crystalloid is influenced by factors such as body weight, surgical duration, other patient characteristics, and unilateral versus bilateral lung transplantation, we focused on the proportion of albumin in the total non-blood intravenous fluid administered. To standardize differences in albumin concentration (5% and 25%), we calculated the corrected 25% albumin proportion (cAP) as follows:

cAP (%) = 100 × (25% albumin + (5% albumin × 0.2))/(25% albumin + 5% albumin + Crystalloid)

Fluid volumes were measured in mL. Here, 100 mL of 5% albumin was equated to 20 mL of 25% albumin and 80 mL of crystalloid based on relative oncotic strength.

Outcomes and Statistical Analysis: The primary outcomes were PGD at 24, 48, and 72 h post-transplant (PGD-24, PGD-48, PGD-72). PGD was analyzed as a binary outcome (presence vs. absence of any PGD). Secondary outcomes included total intravenous fluid (sum of total crystalloid and albumin) per weight (mL/kg), intensive care unit (ICU) length of stay, mechanical ventilation duration, 30-day acute kidney injury (AKI), 30-day mortality, and 1-year mortality. For this regression analysis, total intravenous fluid was normalized to body weight (mL/kg). Binary logistic regression was performed to assess the association of cAP and PGD, total intravenous fluid and PGD, as well as cAP and binary secondary outcomes (30-day AKI, 30-day mortality, and 1-year mortality). Pearson correlation analysis was performed to assess cAP and total intraoperative fluid, ICU length of stay, and mechanical ventilation duration. As a subgroup analysis, we performed the same binary logistic regression on cAP and PGD for those who did and did not receive perioperative extracorporeal membrane oxygenation (ECMO) treatment separately.

## 3. Results

### 3.1. Patient Selection and Characteristics

We identified 216 eligible patients and excluded 26 patients (3 for retransplant, 20 for dual-organ transplant, and 3 for incomplete intraoperative data), yielding a total of 190 patients for analysis. Baseline characteristics are summarized in Table 1. A total of 152 patients underwent bilateral lung transplantation and 38 underwent unilateral transplantation. 124 (65%) patients received ECMO during the perioperative period, 121 of which required intraoperative ECMO or cardiopulmonary bypass. Median total crystalloid given was 2000 mL, which was composed of lactated Ringer’s solution (median 2000 mL) and normal saline (median 400 mL), and the median total albumin given was 200 mL intraoperatively. Median cAP was 9.1%, indicating 9.1% of total intravenous fluid was given as 25% albumin or equivalent oncotic strength of 5% albumin. 120 patients (63%) developed PGD at some timepoint. 119 (62%) patients developed AKI within 30 days. Median ICU length of stay was 11 days, and median duration of mechanical ventilation was 2 days. 13 patients had missing data on the exact duration of mechanical ventilation and were therefore excluded from the analysis of this specific outcome. The 30-day mortality was 1% and the 1-year mortality was 4%. No adverse reactions to albumin were observed in any patient during the study period.

### 3.2. Primary Outcome

As shown in Table 2, higher cAP tended to be associated with slightly reduced odds of PGD, although these associations did not reach statistical significance (PGD-24: OR 0.97, 95% Confidence Interval (CI) 0.93–1.02 *p* = 0.29; PGD-48: OR 1.00, 95% CI 0.95–1.04, *p* = 0.88; PGD-72: OR 0.97, 95% CI 0.93–1.02, *p* = 0.27). Total intravenous fluid was significantly associated with higher odds of PGD-72 (OR 1.02, 95% CI 1.00–1.03, *p* = 0.04), although the magnitude of this association was small. No significant association was found between total intravenous fluid and PGD at other timepoints (PGD-24: OR 1.01, 95% CI 1.00–1.03, *p* = 0.13; PGD-48: OR 1.01, 95% CI 0.99–1.02, *p* = 0.22). Table 3 shows the subgroup analysis of cAP and PGD for those who received perioperative ECMO treatment (ECMO group, *n* = 124) and those who did not (non-ECMO group, *n* = 66). There was no significant association between cAP and PGD in either group (ECMO group: PGD-24: OR 0.99 95% CI 0.93–1.04 *p* = 0.67; PGD-48: OR 0.98, 95% CI 0.93–1.04 *p* = 0.51; PGD-72: OR 0.98, 95% CI 0.92–1.04 *p* = 0.45. non-ECMO group: PGD-24: OR 0.95, 95% CI 0.86–1.04, *p* = 0.24; PGD-48: OR 1.04, 95% CI 0.95–1.13, *p* = 0.41; PGD-72: OR 0.95, 95% CI 0.87–1.04, *p* = 0.29).

### 3.3. Secondary Outcome

cAP demonstrated a weak negative correlation with total intraoperative fluid (*r* = −0.15, *p* = 0.03), indicating that substituting albumin for crystalloid decreases total intraoperative fluid. No significant correlation was found between cAP and ICU length of stay (*r* = −0.10, *p* = 0.17) and mechanical ventilation duration (*r* = −0.09, *p* = 0.26). Similarly, no significant association was found between cAP and 30-day AKI (OR 1.00, 95% CI 0.96–1.05, *p* = 0.92), 30-day mortality (OR 0.78, 95% CI 0.78 0.58–1.05, *p* = 0.10), or 1-year mortality (OR 0.92, 95% CI 0.81–1.05, *p* = 0.21) (Table 4).

## 4. Discussion

Although albumin substitution is commonly practiced in cardiothoracic surgery, its clinical utility remains controversial [9,10]. Albumin has been thought to reduce capillary leak and prolong intravascular volume retention, and these theoretical benefits translated into reduced total intravenous fluid in our study. This supports the notion that albumin may allow us to achieve hemodynamic goals with lower total intravenous fluid. We observed a small but statistically significant association between total intravenous fluid and PGD-72, which aligns with prior studies suggesting an association between larger intravenous fluid and PGD. Although the odds ratio per unit increase was small (OR 1.02 per mL/kg), this represents a roughly 2% increase in PGD odds for each 1 mL/kg of intravenous fluid. For a 70 kg recipient, an additional 1 L of fluid (14 mL/kg) could translate to about a 28% increase in risk of PGD, underscoring that even small per-unit effects may become clinically meaningful over larger fluid volumes. However, cAP itself was not independently associated with a reduction in PGD. Other reported risk factors for PGD, such as age, BMI, cardiac index, pulmonary hypertension, preoperative oxygen requirements, use of vasoactive medications, and donor characteristics [11,12] may have confounded or overshadowed the potential benefits of albumin. Moreover, postoperative fluid management in the ICU, which we did not analyze in this study, may have influenced the development of PGD. We observed a 22% reduction in the odds of 30-day mortality, but this did not reach statistical significance. The lack of association between albumin use and improved secondary outcomes, such as 30-day AKI, mechanical ventilation duration, ICU length of stay, or short-term mortality, aligns with existing evidence, which has failed to demonstrate a meaningful clinical benefit of albumin [13]. We introduced the cAP metric, which standardizes albumin exposure across different concentrations (5% and 25%) and may serve as a reproducible tool for future research. This approach attempts to isolate the effect of albumin from total intravenous fluid, thereby addressing the confounding inherent to fluid management studies. This metric remains a simplified and unvalidated approximation and should be interpreted cautiously. Furthermore, several limitations must be considered. This is an exploratory, hypothesis-generating study, and the nature of a retrospective single-center design limits the causal inference. The small sample size (*n* = 190) has underpowered our study to detect the small effect size of PGD reduction, as well as rare outcomes such as mortality. Post hoc power analysis revealed low statistical power (25% for PGD-24 and PGD-72, and 5% for PGD-48 at α = 0.05), indicating that the nonsignificant associations between cAP and PGD may have been due to insufficient sample size rather than a true lack of effect. The subgroup analysis on ECMO utilization was also limited by statistical power. Decisions regarding fluid management, including albumin administration, as well as ECMO utilization, were not institutionally standardized and may reflect clinician preference, which may further confound causal inference. Large, prospective, ideally randomized studies are warranted to evaluate the role of albumin in lung transplantation fluid management.

## 5. Conclusions

Intraoperative albumin substitution during lung transplantation modestly reduced total intravenous fluid administration but was not independently associated with significant reductions in PGD or improvements in other short-term outcomes.

## Figures and Tables

**Table 1:** Patient characteristics.

Age	63 (54–68) years *	
Biological sex	126 male, 64 female	
BMI	26 (22–29) kg/m^2^ *	
Preoperative cardiac index on RHC	3.2 (2.6–3.9) L/min/m^2^ *	excluding 75 patients with missing data
Preoperative mean PA pressure	25 (20–30) mmHg *	excluding 66 patients with missing data
Lung transplantation type	Unilateral 38, Bilateral 152	
Preoperative ECMO use	None 169, VV 16, VA 5	
Intraoperative ECMO use	None 69, VV 18, VA 95, CPB 8	
Postoperative ECMO use	None 161, VV 22, VA 7	
Corrected Albumin Proportion (cAP) **	9.1 (5.2–13) % *	
Intraoperative total crystalloid	2000 (1500–2900) mL *	
Intraoperative LR	2000 (1500–2500) mL *	
Intraoperative NS	400 (238–500) mL *	
Intraoperative total albumin	200 (150–350) mL *	
Intraoperative 25% albumin	200 (200–300) mL *	
Intraoperative 5% albumin	1000 (500–1375) mL *	
Intraoperative PRBC	250 (0–502) mL *	
Intraoperative non-PRBC blood product	0 (0–675) mL *	
Intraoperative total blood product	500 (0–1338) mL *	
PGD-24	None: 100, grade 1: 23, grade 2: 23, grade 3: 44	
PGD-48	None: 85, grade 1: 25, grade 2: 37, grade 3: 43	
PGD-72	None: 102, grade 1: 22, grade 2: 27, grade 3: 39	
30-day AKI	119 (62%)	
ICU length of stay	11 (5–21) days *	
Duration of mechanical ventilation	2 (1–5) days *	excluding 13 patients with missing data
30-day mortality	3 (1%)	
1-year mortality	8 (4%)	

* Median (Interquartile Range (IQR)). ** cAP (%) = 100 × (25% albumin + (5% albumin × 0.2))/(25% albumin + 5% albumin + Crystalloid). Unit for fluid is mL. Abbreviation: BMI: Body Mass Index; RHC: Right Heart Catheterization; PA: Pulmonary Artery; ECMO: Extracorporeal Membrane Oxygenation; VV: veno-venous; VA: veno-arterial; LR: Lactated Ringer’s; NS: Normal Saline; PRBC: Packed Red Blood Cells; PGD: Primary Graft Dysfunction; AKI: Acute Kidney Injury; ICU: Intensive Care Unit.

**Table 2:** Total intraoperative fluid (mL/kg) and PGD.

cAP (%)			
PGD Timepoint	Odds Ratio	95% CI	*p*-Value
PGD-24	0.97	0.93–1.02	0.29
PGD-48	1.00	0.95–1.04	0.88
PGD-72	0.97	0.93–1.02	0.27
**Total Intravenous Fluid (mL/kg)**			
**PGD Timepoint**	**Odds Ratio**	**95% CI**	***p*-Value**
PGD-24	1.01	1.00–1.03	0.13
PGD-48	1.01	0.99–1.02	0.22
PGD-72	1.02	1.00–1.03	0.04

Abbreviation: PGD, Primary Graft Dysfunction; cAP, corrected Albumin Proportion (%); CI, Confidence Interval.

**Table 3:** cAP (%) and PGD in the ECMO and non-ECMO patient group.

ECMO Group (*n* = 124)			
PGD Timepoint	Odds Ratio	95% CI	*p*-Value
**PGD-24**	0.99	0.93–1.04	0.67
**PGD-48**	0.98	0.93–1.04	0.51
**PGD-72**	0.98	0.92–1.04	0.45
**Non-ECMO Group (*n* = 66)**			
**PGD Timepoint**	**Odds Ratio**	**95% CI**	***p*-Value**
**PGD-24**	0.95	0.86–1.04	0.24
**PGD-48**	1.04	0.95–1.13	0.41
**PGD-72**	0.95	0.87–1.04	0.29

Abbreviation: cAP, corrected Albumin Proportion (%); PGD, Primary Graft Dysfunction; ECMO, Extracorporeal Membrane Oxygenation; CI, Confidence Interval.

**Table 4:** cAP (%) and postoperative outcomes.

Outcome	Correlation Coefficient		*p*-Value
**Total intravenous fluid (mL/kg)**	−0.15		0.03
**ICU length of stay (days)**	−0.10		0.17
**Mechanical ventilation duration (days)**	−0.09		0.26
	**Odds Ratio**	**95% CI**	***p*-Value**
**30-day AKI**	1.00	0.96–1.05	0.92
**30-day mortality**	0.78	0.58–1.05	0.10
**1-year mortality**	0.92	0.81–1.05	0.21

Abbreviation: cAP, corrected Albumin Proportion (%); ICU, Intensive Care Unit; AKI, Acute Kidney Injury; CI, Confidence Interval.

## Data Availability

Data is unavailable due to privacy restrictions.
